# Enhanced Expression of TRIM46 in Ovarian Cancer Cells Induced by Tumor-Associated Macrophages Promotes Invasion via the Wnt/β-Catenin Pathway

**DOI:** 10.3390/cells14030214

**Published:** 2025-02-02

**Authors:** Yi-Yue Wang, Min-Jun Choi, Jin-Hyung Kim, Jung-Hye Choi

**Affiliations:** 1 Guizhou Provincial Engineering Technology Research Center for Chemical Drug R&D, State Key Laboratory of Discovery and Utilization of Functional Components in Traditional Chinese Medicine & School of Pharmaceutical Sciences, Guizhou Medical University, Guiyang 561113, China; wangyiyue@gmc.edu.cn; 2 Department of Biomedical and Pharmaceutical Science, College of Pharmacy, Kyung Hee University, Seoul 02447, Republic of Korea; ideanaeda@khu.ac.kr (M.-J.C.); jenon@khu.ac.kr (J.-H.K.)

**Keywords:** tumor-associated macrophages, ovarian cancer, invasion, TRIM46, Wnt/β-catenin

## Abstract

Metastasis presents significant challenges in ovarian cancer treatment. Tumor-associated macrophages (TAMs) within the tumor microenvironment (TME) facilitate metastasis through epithelial-mesenchymal transition, yet the molecular underlying mechanisms are not fully understood. Here, we identified that tripartite motif-containing 46 (TRIM46) is significantly upregulated in ovarian cancer cells treated with a conditioned medium derived from macrophages stimulated by ovarian cancer cells (OC-MQs). Furthermore, TRIM46 was highly expressed in late-stage ovarian cancer patients and was associated with poor prognosis. Silencing of TRIM46 suppressed cancer cell invasion stimulated by OC-MQ and mesenchymal marker expression without affecting cell viability. Gene set enrichment analysis showed that the Wnt/β-catenin pathway is enriched in the high-TRIM46 expression group. Importantly, the inhibition of TRIM46-mediated β-catenin nuclear translocation and ovarian cancer cell invasion was reversed by CHIR99021, a Wnt/β-catenin activator. Additionally, C-X-C motif chemokine ligand 8 (CXCL8) was identified as being highly expressed in peritoneal MQs from the ascites of ovarian cancer patients and was positively correlated with C-X-C chemokine receptor 1/2 (CXCR1/2) expression in tumor cells. Notably, pre-treatment with reparixin, a CXCR1/2 inhibitor, blocked OC-MQ-induced TRIM46 expression and cell invasion. These results suggest that CXCL8 derived from TAMs promotes human ovarian cancer cell invasion via the Wnt/β-catenin pathway by upregulating TRIM46.

## 1. Introduction

Ovarian cancer is one of the most lethal gynecological cancers worldwide. The 5-year survival rate for patients with stage III ovarian cancer patients is approximately 40%, while for those with stage IV ovarian cancer patients, it is only 20% [1]. This is largely due to the fact that the early symptoms of ovarian cancer are subtle, which results in most cases being diagnosed at advanced stages with widespread metastasis. This metastatic progression is a major cause of unfavorable outcomes and increased mortality in affected individuals [2]. Thus, there is an urgent need to elucidate the molecular mechanisms driving ovarian cancer metastasis.

Ovarian cancer metastasis is intricately linked to the tumor microenvironment (TME), which plays a critical role in facilitating tumor dissemination and progression. The TME comprises various non-malignant cells, including stromal cells, immune cells, and components of the extracellular matrix, which interact dynamically with the cancer cells. These interactions contribute to tumorigenesis and progression, influencing ovarian cancer cell proliferation and invasion [3]. Soluble factors such as cytokines and chemokines secreted in the TME can enhance the invasive potential of ovarian cancer cells through processes such as epithelial-mesenchymal transition (EMT) and the creation of a supportive niche for metastatic growth [4]. These findings highlight potential therapeutic targets for ovarian cancer.

Among TME components, macrophages (MQs) originating from peripheral blood monocytes infiltrate the TME and differentiate into tumor-associated macrophages (TAMs) [5]. Previous studies have shown that TAMs are crucial in ovarian cancer progression, promoting cell proliferation, metastasis, angiogenesis, and immunosuppression [6]. Despite extensive studies on TAM-mediated tumor invasion, the specific gene expression changes and mechanisms driving TAM-induced ovarian cancer cell invasion remain unclear.

The tripartite motif (TRIM) protein family, characterized by a conserved RING finger, B-box, and coiled-coil domain, contributes significantly to cellular functions such as immune regulation, growth, and differentiation. Several TRIM proteins are implicated in cancer progression, functioning as either oncogenes or tumor suppressors, depending on the context [7]. TRIM proteins have been linked to EMT regulation, a hallmark of metastasis [8]. TRIM46, previously recognized for its role as a microtubule fascicle organizer in the proximal axon [9], has demonstrated a significant impact on the proliferation and migration of breast cancer cells [10]. Nevertheless, the function of TRIM46 in ovarian cancer remains largely uninvestigated.

In this study, we examined the gene expression patterns of ovarian cancer cells stimulated by macrophages and identified TRIM46 as a key player in TAM-facilitated invasion. We further explored the underlying molecular mechanisms, focusing on the involvement of TRIM46 and its regulation via the Wnt/β-catenin pathway.

## 2. Materials and Methods

### 2.1. Materials

The MTT reagent (3-(4,5-dimethylthiazol-2-yl)-2,5-diphenyl tetrazolium bromide) was purchased from Thermo Fisher Scientific (Waltham, MA, USA). Polycarbonate Transwell inserts with 8-µm pore-sized filters, free from polyvinylpyrrolidone, and BD Matrigel™ Basement Membrane Matrix were purchased from BD Biosciences in San Jose, CA, USA. Small interfering RNA (siRNA) targeting TRIM46, along with primers for TRIM46, SNAIL, CDH2, FN, VIM, ZEB1, and β-actin, were obtained from Bioneer Technology, located in Seoul, Republic of Korea. Supplementary Table S1 contains a detailed list of primer sequences. CHIR99021 was supplied by Selleck Chemicals LLC (Houston, TX, USA), while phorbol myristate acetate (PMA) and a 1% crystal violet solution were acquired from Sigma-Aldrich (St. Louis, MO, USA).

### 2.2. Cell Culture and Preparation of Conditioned Medium

Human ovarian cancer cell lines A2780, SKOV3, ES2, and human monocytic cell line THP-1 were originally from the American Type Culture Collection (ATCC; Manassas, VA, USA). All ovarian cancer cell lines were cultured in RPMI 1640 medium supplemented with 5–10% FBS, 100 U/mL of penicillin, and 100 μg/mL of streptomycin. THP-1 cells were differentiated into macrophages (MQs) by exposing them to phorbol 12-myristate 13-acetate (PMA) for 24 h. Cultures were maintained at 37 °C in a humidified atmosphere containing 5% CO_2_. Macrophages were activated into OC-MQs by incubating them with a conditioned medium derived from ovarian cancer cell lines for 24 h.

Conditioned medium (CM) was prepared by culturing ovarian cancer cells, MQs, and OC-MQs in 100-mm dishes filled with 10 mL of fresh culture medium for 24 h. After incubation, the media were collected and centrifuged at 2500 rpm for 7 min to eliminate cell debris. The clarified supernatant was subsequently stored at −80 °C for future analysis.

### 2.3. Invasion Assay

Cell invasion ability was assessed using Matrigel-coated Transwell inserts. Briefly, polycarbonate filters of the inserts were coated with a Matrigel matrix layer and incubated for 4 h. Cells were seeded into the upper chambers in medium containing 1% FBS, while the lower chambers were filled with medium supplemented with 5% FBS. Following 48 h of incubation at 37 °C, filters were stained with a 0.05% crystal violet solution. The invaded cells were visualized under a light microscope, and three random fields per sample were analyzed at 200× magnification. The percentage of invading cells was quantified using ImageJ software (NIH, Bethesda, MD, USA).

### 2.4. Cell Viability Assay

The cells underwent transfection with TRIM46 siRNA or control siRNA for 24 h, after which they were trypsinized and plated in 96-well plates. After overnight incubation, the cells were exposed to TAM-CM for 24 h. Subsequently, an MTT solution (prepared as a 1 mg/mL stock) was added to each well, and the plates were incubated for 4 h. The formazan crystals formed in the wells were dissolved in DMSO, and absorbance was measured at 540 nm using a microplate spectrophotometer (SpectraMax, Molecular Devices, Sunnyvale, CA, USA).

### 2.5. mRNA Sequencing

For transcriptomic profiling, ovarian cancer cells were treated with either plain medium (PM) or MQ-CM. RNA sequencing was conducted by eBiogen, Inc. (Seoul, Republic of Korea) in accordance with the methodology described in our previous study [11]. Sequencing libraries were prepared from 500 ng of RNA. The sequencing reads were mapped using Bowtie 2, v2.4.2, and the identification of differentially expressed genes was carried out with Bedtools. The RT data underwent global normalization with Genowiz^TM^ software version 4.0.5.6 (Ocimum Biosolutions, Hyderabad, India).

### 2.6. Bioinformatics Analysis

To evaluate the role of TRIM46 in metastasis, we analyzed the GSE9891 dataset from the Gene Expression Omnibus (GEO) database. Kaplan–Meier survival plots for overall survival and progression-free survival were generated using the KM-Plotter platform (https://kmplot.com/), accessed on 2 September 2024, based on TRIM46 expression data from a cohort of 1435 ovarian cancer cases provided by GEO and TCGA datasets available on the platform. High and low expression groups were determined using the median as a threshold. The hazard ratio (HR) with a 95% confidence interval (CI) and log-rank *p*-values were calculated. Additionally, gene set enrichment analysis (GSEA) was performed on the GSE26193 dataset, categorizing ovarian tumor samples into high and low TRIM46 expression groups based on the median value. Enrichment was analyzed using HALLMARK gene sets in the GSEA software, with statistical significance defined as *p* < 0.05.

### 2.7. Real-Time RT-PCR

For RNA isolation, the Easy-BLUE^TM^ Total RNA Extraction Kit (iNtRON Biotechnology Inc., Seoul, Republic of Korea) was used, and cDNA synthesis was achieved via the TOPscriptTM RT DryMIX kit (Enzynomics, Daejeon, Republic of Korea). Quantitative real-time PCR (qRT-PCR) was carried out using the SYBR Premix Ex Taq™ Kit (TaKaRa, Kyoto, Japan). The relative mRNA expression levels were calculated using the comparative cycle threshold (CT) method, normalizing β-actin as the reference gene.

### 2.8. Western Blot Analysis

Cell pellets were rinsed twice with cold PBS and lysed in protein extraction buffer (iNtRON Biotechnology, Inc., Seoul, Republic of Korea). For nuclear extraction, cell pellets were first washed with PBS and then resuspended in a hypotonic buffer composed of 10 mM N-2-hydroxyethylpiperazine-N′−2-ethanesulfonic acid (HEPES, pH 7.9), 1.5 mM MgCl_2_, 10 mM KCl, 0.2 mM PMSF, 0.5 mM DTT, and 10 μg/mL aprotinin. This mixture was incubated on ice for 15 min. Cells were lysed by adding 0.1% Nonidet P-40, followed by vigorous vortexing for 10 s. The samples were then centrifuged at 12,000× *g* for 1 min at 4 °C. The supernatant was discarded, and the nuclear pellets were resuspended in a high-salt buffer containing 20 mM HEPES (pH 7.9), 400 mM KCl, 25% glycerol, 1.5 mM MgCl_2_, 0.2 mM EDTA, 0.5 mM DTT, 1 mM NaF, and 1 mM sodium orthovanadate. Proteins were resolved on 8–12% SDS-polyacrylamide gels, transferred to PVDF membranes, and blocked before incubation with specific primary antibodies overnight at 4 °C. After washing with TBST, membranes were treated with HRP-conjugated secondary antibodies for 2 h. Detection was performed using the ECL chemiluminescence system, and ImageJ software was used for densitometric analysis. Each experiment was performed with at least three independent replicates, and the western blot bands shown are representative images.

### 2.9. Statistical Analysis

All data are presented as the mean ± SD. Statistical analyses were performed using GraphPad Prism software (GraphPad Software, Inc., La Jolla, CA, USA). One-way ANOVA, two-way ANOVA, or Student’s *t*-test was employed as appropriate, with *p* < 0.05 considered statistically significant.

## 3. Results

### 3.1. TRIM46 Is Upregulated in Macrophage-Stimualted Ovarian Cancer Cells and Correlates with Poor Prognosis

**Figure 1:**
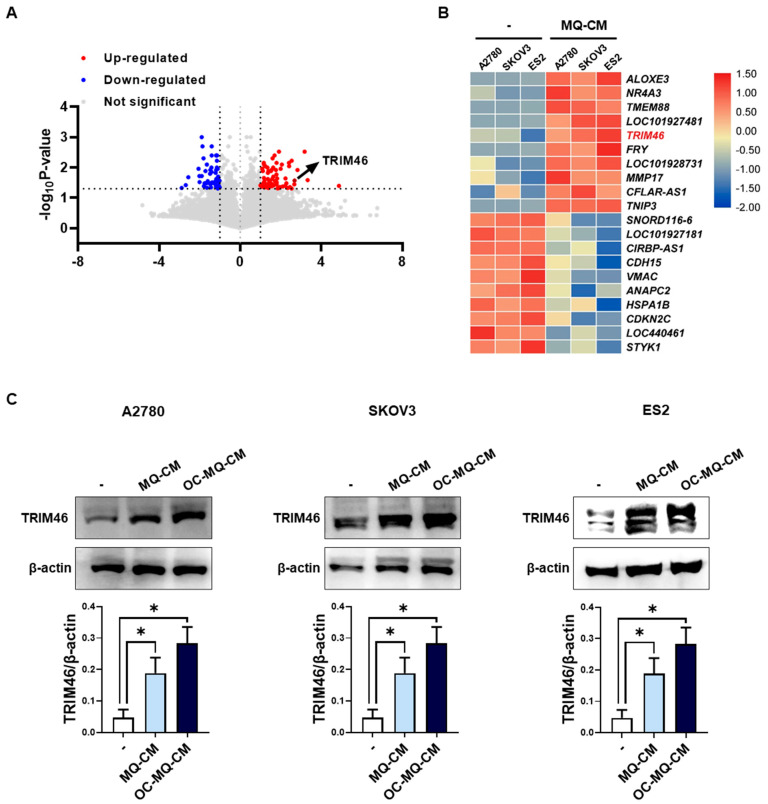
Upregulation of TRIM46 in OC-MQ-stimulated ovarian cancer cells. (**A**) A volcano plot displaying differentially expressed genes (DEGs) (fold change > 2, *p*-value < 0.05) in ovarian cancer cells (A2780, SKOV3, and ES2) stimulated with conditioned medium (CM) from macrophages (MQs) (MQ-CM) compared to untreated cells (**B**) A heatmap showing the top 10 upregulated and downregulated DEGs between MQ-induced and untreated ovarian cancer cells. (**C**) The protein expression levels of TRIM46 in ovarian cancer cells, stimulated with MQ-CM or CM from cancer-stimulated macrophages (OC-MQ-CM) for 24 h, were assessed by western blotting. Data are representative of at least three independent experiments. * *p* < 0.05.

**Figure 2:**
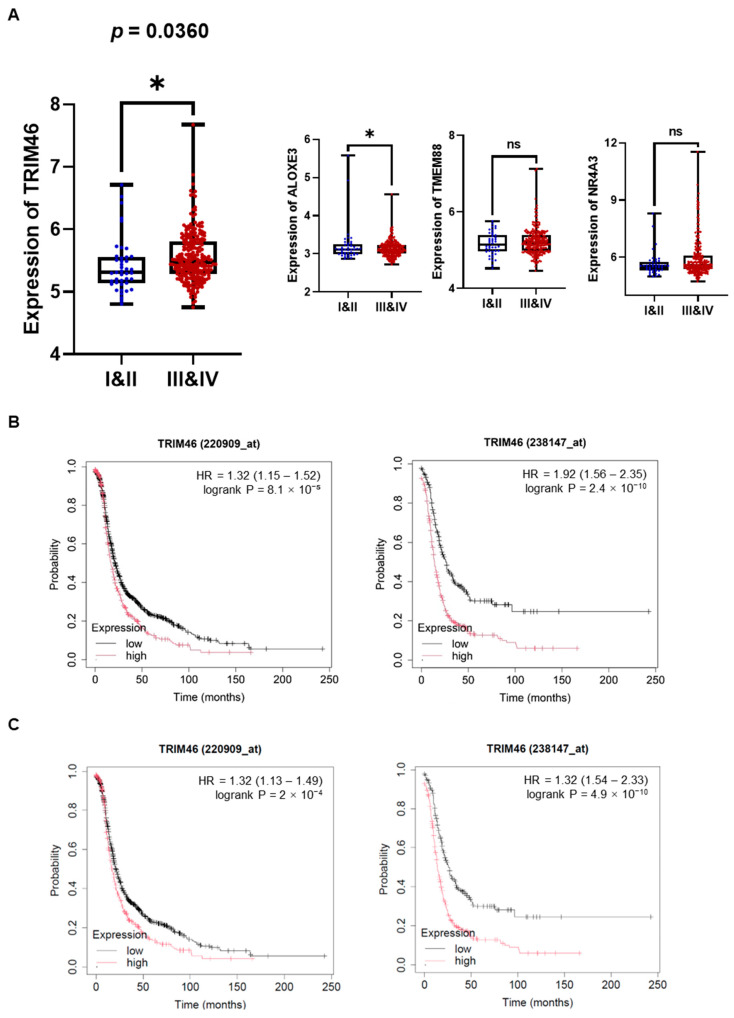
Enhanced expression of TRIM46 in patients with advanced ovarian cancer and its association with poor prognosis. (**A**) The expression levels of TRIM46, ALOXE3, NR4A3, and TMEM88 in tumor tissues from patients with stage I & II (n = 42) and stage III & IV (n = 243) ovarian cancer were analyzed using the GSE9891 dataset. (**B**,**C**) The prognostic value of TRIM46 in ovarian cancer patients, in terms of overall survival (**B**) and progression-free survival (**C**). was evaluated using the Kaplan-Meier Plotter. * *p* < 0.05, ns indicates no significant difference.

Macrophages in the tumor microenvironment (TME) are widely recognized for interacting with ovarian cancer cells by secreting soluble factors that reprogram cancer cells to adopt a pro-invasive phenotype [12]. To gain deeper insights into the molecular mechanisms underlying macrophage-induced ovarian cancer cell invasion, we performed mRNA sequencing to detect differentially expressed genes (DEGs) in human ovarian cancer cells (A2780, SKOV3, ES2) stimulated with macrophage-derived conditioned medium (MQ-CM). Volcano plots revealed significant gene expression changes between ovarian cancer cells treated with MQ-CM and those exposed to plain medium (Figure 1A and Supplementary Table S2), with the top upregulated and downregulated genes displayed in Figure 1B. Among the upregulated genes, *TRIM46*, a member of the tripartite motif (TRIM) protein family implicated in cancer development and progression [8], was notably up-regulated in macrophage-stimulated ovarian cancer cells. Furthermore, treatment with MQ-CM or cancer-stimulated macrophage CM (OC-MQ-CM) significantly increased TRIM46 protein expression (Figure 1C). To explore the clinical significance of TRIM46, we analyzed publicly available datasets and found that TRIM46 expression was significantly higher in advanced-stage ovarian cancer tissues compared to early-stage disease. Genes located above TRIM46 in the heatmap, such as *ALOXE3*, *NR4A3*, and *TMEM88*, did not exhibit similar upregulation in advanced-stage cancer tissues (Figure 2A). Survival analysis indicated that elevated TRIM46 expression correlated with reduced overall survival and progression-free survival in ovarian cancer patients (Figure 2B,C). These results collectively imply that TRIM46 expression is regulated by macrophage-derived in the TME and may contribute to ovarian cancer metastasis.

### 3.2. TRIM46 Is Involved in OC-MQ-Induced Ovarian Cancer Cell Invasion via Epithelial-to-Mesenchymal Transition

**Figure 3:**
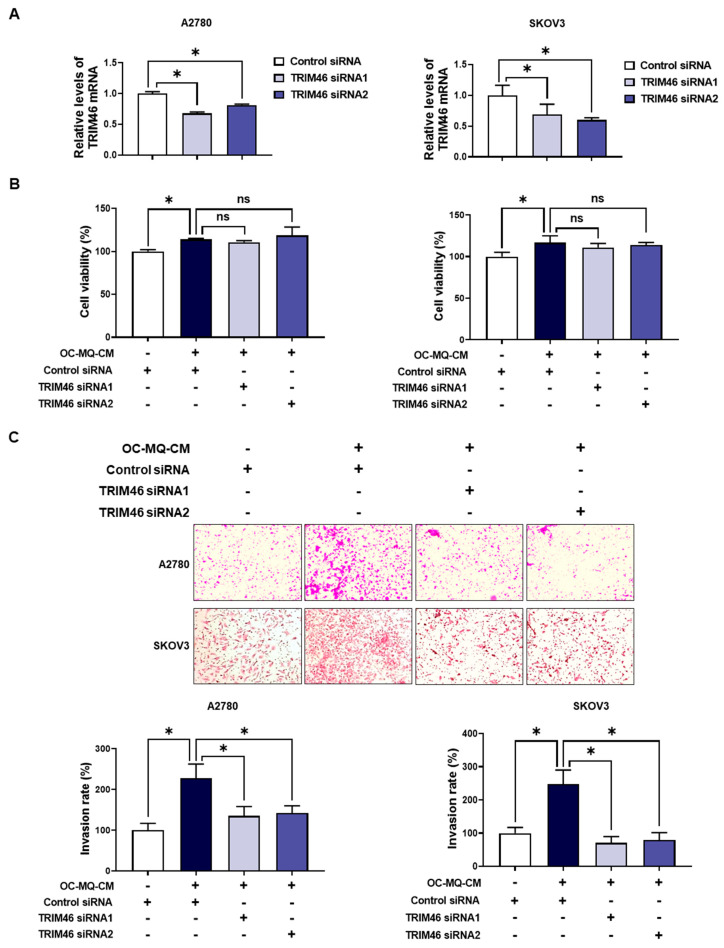
Involvement of TRIM46 in OC-MQ-facilitated ovarian cancer cell invasion. (**A**) A2780 and SKOV3 cells were transfected with TRIM46 siRNAs (10 nM) or control siRNA for 24 h. The knockdown efficiency of TRIM46 siRNAs was confirmed by real-time RT-PCR. (**B**) Ovarian cancer cells, following transfection with TRIM46 siRNA or control siRNA, were cultured in the conditioned medium of cancer-stimulated macrophages (OC-MQ-CM) for 24 h. The cell viability was measured by MTT assay. (**C**) Ovarian cancer cells, following transfection with TRIM46 siRNA or control siRNA for 24 h, were resuspended in the conditioned medium of OC-MQ-CM. The cells were then placed into Matrigel-coated upper chambers, where they were left to invade over a 24-h period. Data are representative of at least three independent experiments. * *p* < 0.05, ns indicates no significant difference.

**Figure 4:**
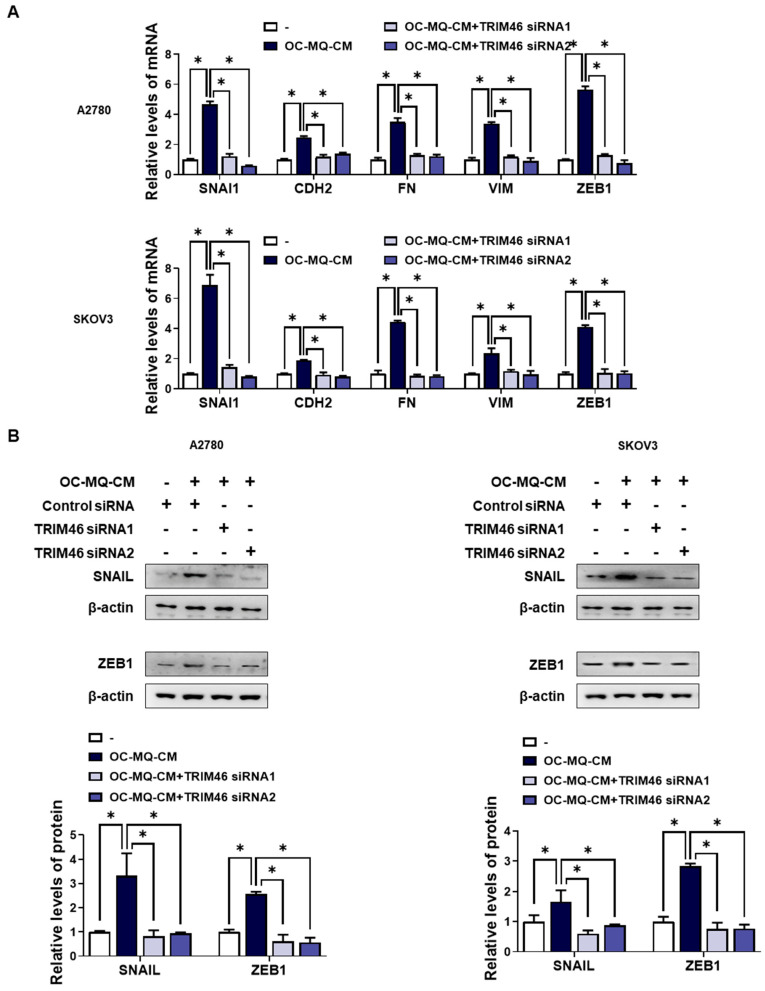
Involvement of TRIM46 in enhanced expression of mesenchymal makers in human ovarian cancer cells stimulated by OC--MQs. A2780 and SKOV3 cells were transfected with TRIM46 siRNAs (10 nM) or control siRNA for 24 h, and subsequently cultured in the conditioned medium of cancer-stimulated macrophages (OC-MQ-CM) for 24 h. (**A**) The mRNA levels of mesenchymal markers were analyzed by real-time RT-PCR. (**B**) The protein levels of mesenchymal markers were determined by western blotting. Data are representative of at least three independent experiments. * *p* < 0.05.

To determine whether the upregulation of TRIM46 in OC-MQ-facilitated ovarian cancer cells contributes to cell invasion, we performed an invasion assay following TRIM46 knockdown using siRNAs. Conditioned medium derived from OC-MQs markedly increased the invasiveness of three ovarian cancer cell lines (Supplementary Figure S1), consistent with previous studies [13,14]. TRIM46 knockdown efficiency was confirmed via RT-PCR (Figure 3A). Importantly, silencing TRIM46 did not affect the cell viability of the cancer cells (Figure 3B). Notably, the suppression of TRIM46 expression substantially reduced OC-MQ-facilitated cancer cell invasion (Figure 3C). EMT, a key process in cancer metastasis [15], was examined for its association with TRIM46. OC-MQs increased mesenchymal markers’ expression, including SNAI1, CDH2, FN, VIM, and ZEB1, in ovarian cancer cells (Figure 4A,B). TRIM46 has been reported to regulate EMT in human renal proximal tubular epithelial cells [16]. In ovarian cancer cells, TRIM46 knockdown suppressed the OC-MQ-induced upregulation of mesenchymal marker mRNA (Figure 4A). Furthermore, the protein expression of EMT markers SNAIL and ZEB1 was reduced following TRIM46 silencing (Figure 4B). These findings demonstrate that TRIM46 mediates TAM-induced ovarian cancer cell invasion via EMT.

### 3.3. Wnt/β-Catenin Signaling Functions as a Downstream Pathway of TRIM46

**Figure 5:**
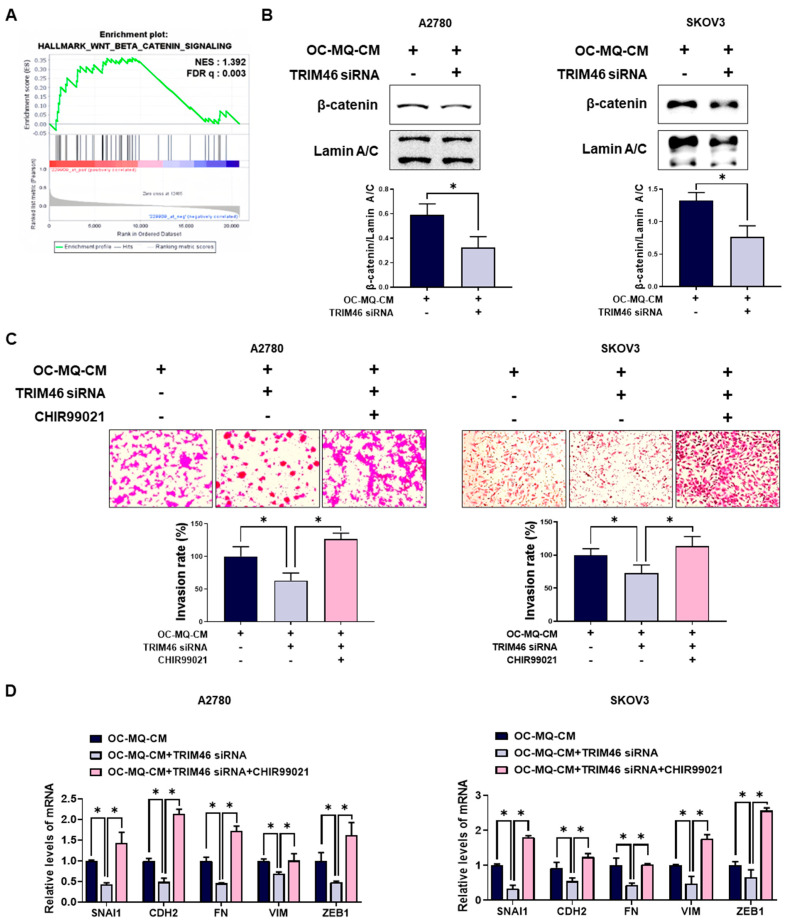
Involvement of the Wnt/β-catenin pathway in TRIM46-stimulated cancer cell invasion. (**A**) Gene Set Enrichment Analysis (GSEA) was performed to assess the enrichment of Wnt/β-catenin signaling-related gene sets in patient tissues with high TRIM46 expression using the GSE26193 dataset. NES: normalized enrichment score; FDR q: false discovery rate q-value. (**B**–**D**) A2780 and SKOV3 cells were transfected with TRIM46 siRNA (10 nM) or control siRNA for 24 h, then cultured in the conditioned medium of cancer-stimulated MQs (OC-MQ-CM) and treated with CHIR99021 (Wnt/β-catenin activator; 6 μM) for an additional 24 h. (**B**) Western blot analysis was conducted to measure β-catenin levels in the nuclear fraction of cell lysates. Nuclear protein lamin A/C was used as a loading control. (**C**) The invasive capacity of ovarian cancer cells was assessed by Transwell assay. (**D**) The mRNA expression levels of mesenchymal markers were quantified by real-time RT-PCR. Data are representative of at least three independent experiments. * *p* < 0.05.

To clarify the mechanism by which TRIM46 stimulates enhanced ovarian cancer cell invasion, we performed a gene set enrichment analysis (GSEA). As shown in Figure 5A, GSEA revealed a significant enrichment of the Wnt/β-catenin pathway, which is closely associated with ovarian cancer cells with high TRIM46 expression. Wnt/β-catenin pathway has been reported to be closely associated with ovarian cancer invasion [17]. Knockdown of TRIM46 significantly decreased the nuclear translocation of β-catenin in ovarian cancer cells, suggesting that TRIM46 expression is positively associated with activation of the Wnt/β-catenin pathway (Figure 5B). Treatment with CHIR99021, a Wnt/β-catenin activator, restored both invasion (Figure 5C) and mesenchymal marker expression (Figure 5D) in TRIM46-depleted ovarian cancer cells. These data indicate that TRIM46 promotes ovarian cancer invasion by activating the Wnt/β-catenin pathway.

### 3.4. CXCL8 Derived from TAMs Promotes TRIM46 Expression and Ovarian Cancer Cell Invasion

**Figure 6:**
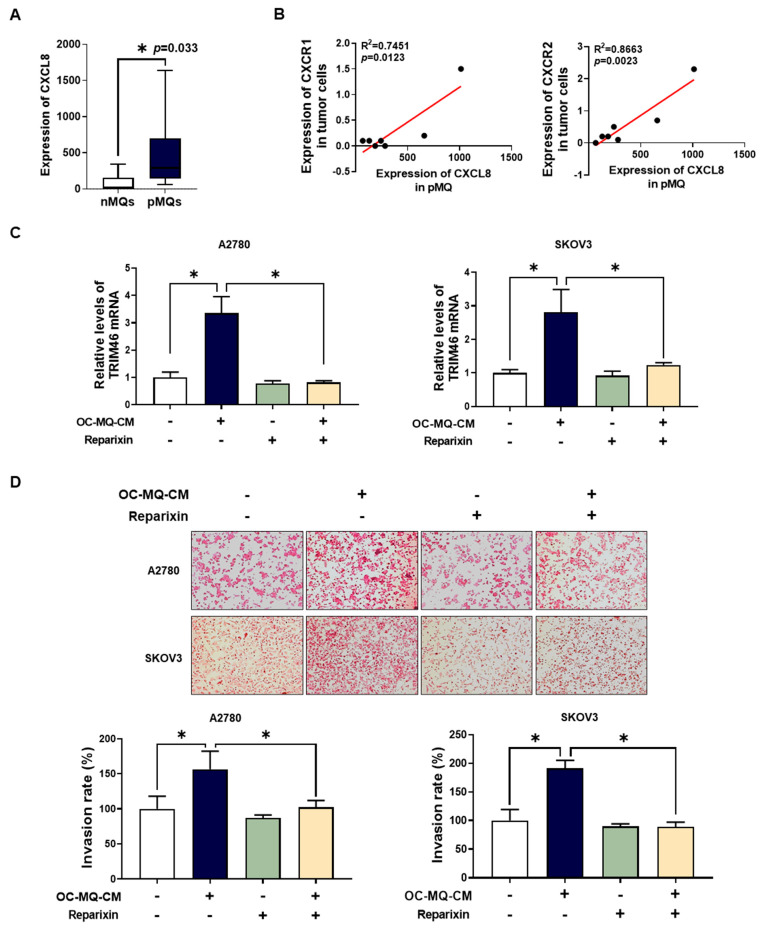
Association of the CXCL8-CXCR1/2 axis with TRIM46 expression of ovarian cancer cells. (**A**) The expression of CXCL8 was analyzed in peritoneal macrophages from non-malignant diseases (n = 7) (nMQs) and from the ascites of ovarian cancer patients (pMQs; n = 16) [19]. (**B**) The correlation between CXCL8 expression in pMQs and the expression of its receptors, CXCR1 and CXCR2, in matched tumor cells from the same patients. (**C**,**D**) Ovarian cancer cells were cultured with the conditioned medium of cancer-stimulated macrophages (OC-MQ-CM) following pre-treatment with reparixin (0.1 μM) for 2 h. After 24-h incubation, (**C**) the mRNA expression level of TRIM46 was analyzed by real-time RT-PCR; (**D**) a transwell assay was performed to evaluate the invasive capacity of ovarian cancer cells. Data are representative of at least three independent experiments. * *p* < 0.05.

Soluble factors such as growth factors, cytokines, and chemokines derived from TAMs play a pivotal role in ovarian cancer metastasis [18]. To identify specific factors in TAM-CM that contribute to ovarian cancer cell invasion, we analyzed publicly available Gene Expression Omnibus (GEO) datasets [19] focused on cytokine expression in TAMs from ovarian cancer ascites. Among these, C-X-C motif chemokine ligand 8 (CXCL8) showed significantly elevated expression in peritoneal MQs (pMQs) from the ascites of ovarian cancer patients (Figure 6A) and macrophages stimulated by ovarian cancer cells (Supplementary Figure S2). CXCL8 has been reported to drive EMT via the Wnt/β-catenin pathway in ovarian cancer cells [20]. Further analysis revealed a positive correlation between CXCL8 expression in pMQs and its receptors, CXCR1 and CXCR2, in matched tumor cells (Figure 6B). In addition, we found that OC-MQs increased CXCR1 and CXCR2 expression, including in ovarian cancer cells (Supplementary Figure S3). To evaluate whether CXCL8-mediated ovarian cancer cell invasion involves TRIM46 upregulation, we pre-treated ovarian cancer cells with reparixin, a CXCR1/2 inhibitor. Reparixin significantly reduced OC-MQ-induced TRIM46 expression (Figure 6C) and suppressed the invasion of ovarian cancer cells (Figure 6D). These results suggest that TAMs promote ovarian cancer cell invasion by upregulating TRIM46 via the CXCL8-CXCR1/2 signaling axis.

## 4. Discussion

TRIM46, a member of the TRIM protein family, has been implicated in various cancers, but its specific role in ovarian cancer was previously unknown. Our results demonstrate for the first time that TAMs enhance TRIM46 expression in ovarian cancer cells. Previous studies on other TRIM family members have shown their oncogenic potential in promoting cancer progression and metastasis. For instance, TRIM52, TRIM37, and TRIM47 have been linked to ovarian cancer invasion [21,22,23], while other members, such as TRIM37 in hepatocellular carcinoma and TRIM24 in colorectal cancer, are known to facilitate metastasis in other cancer types [24,25,26,27]. Similarly, TRIM46 has been shown to promote chemoresistance in lung cancer [28] and enhance osteosarcoma cell survival [29], supporting its role in cancer progression. In this study, TRIM46 knockdown significantly reduced TAM-induced ovarian cancer invasion, underscoring its pro-metastatic function. Furthermore, elevated TRIM46 expression in advanced ovarian cancer patients and its association with poor prognosis suggest that TRIM46 could serve as both a prognostic biomarker and a therapeutic target.

A critical mechanism identified in this study is the involvement of the Wnt/β-catenin signaling pathway in TAM-induced ovarian cancer invasion. The Wnt/β-catenin signaling pathway is a well-established driver of tumor progression, promoting cell proliferation, EMT, and invasion in various cancers, including ovarian cancer [30]. Our findings show that TRIM46 upregulates Wnt/β-catenin signaling, as evidenced by the increased nuclear translocation of β-catenin. Notably, TRIM46 functions as an E3 ubiquitin ligase, a characteristic feature of TRIM proteins [28,31,32]. By targeting components of the β-catenin destruction complex, TRIM46 likely facilitates β-catenin stabilization and activation, thereby driving EMT-associated gene expression and enhancing cancer cell invasiveness. This mechanism is consistent with previous reports of other TRIM proteins, such as TRIM31 in gastric cancer [33] and TRIM8 in hepatocellular carcinoma [34], which promote Wnt/β-catenin activation via E3 ligase-mediated ubiquitination.

In addition to the role of TRIM46, we also explored the upstream signaling pathways that regulate its expression. TAMs secrete a variety of soluble factors, including cytokines and growth factors, that modulate cancer cell behavior [35]. Among these factors, chemokine (C-X-C motif) ligand 8 (CXCL8), also known as interleukin-8 (IL-8), emerged as a key regulator of TRIM46 expression. CXCL8, which is highly expressed in TAMs compared to resident peritoneal macrophages, has been shown to enhance ovarian cancer metastasis through EMT and Wnt/β-catenin signaling [20]. In our study, pre-treatment with reparixin, a CXCR1/2 inhibitor, reduced CXCL8-induced TRIM46 expression and cancer cell invasion, further implicating the CXCL8-CXCR1/2 axis in ovarian cancer metastasis. CXCL8 has also been shown to promote cancer progression through autocrine signaling, reinforcing its importance in ovarian cancer TME [36]. The CXCL8-CXCR1/2 axis represents a promising therapeutic target, particularly given its role in multiple cancer types. Clinical trials targeting this pathway have shown potential in advanced solid tumors, metastatic melanoma, and colorectal carcinoma [37]. In ovarian cancer, therapeutic strategies that inhibit CXCL8 or its receptors could reduce TAM-mediated cancer progression and metastasis. Our findings suggest that such approaches, combined with TRIM46-targeting therapies, could be particularly effective in modulating the pro-invasive TME.

Despite these advancements, several questions remain. While our study identified TRIM46 as a critical mediator of TAM-induced invasion and linked its activity to Wnt/β-catenin signaling, further research is needed to clarify the exact molecular interactions between TRIM46 and the β-catenin destruction complex. Additionally, the broader implications of TRIM46 in chemoresistance and its interplay with other signaling pathways warrant investigation. Exploring how TRIM46 regulates other components of the TME, including stromal cells and immune components, could provide a more comprehensive understanding of its role in ovarian cancer progression.

## 5. Conclusions

In conclusion, this study sheds light on the pro-metastatic role of TRIM46 in ovarian cancer and its regulation by TAMs via the CXCL8-CXCR1/2 axis. Mechanistically, TRIM46 promotes Wnt/β-catenin signaling, enhancing EMT and invasion in ovarian cancer cells. These findings not only expand our understanding of ovarian cancer metastasis but also highlight TRIM46 and the CXCL8-CXCR1/2 axis as potential therapeutic targets. Future efforts should focus on translating these findings into clinical applications, paving the way for novel strategies to combat ovarian cancer progression and metastasis.

## Data Availability

The data underlying the findings of this study can be obtained from the corresponding author upon reasonable request.

## Supplementary Materials

The following supporting information can be downloaded at: https://www.mdpi.com/article/10.3390/cells14030214/s1, Figure S1: Effect of OC-MQ-CM on ovarian cancer cell invasion; Figure S2: Expression levels of CXCL8 in ovarian cancer cells and macrophages stimulated by ovarian cancer cells; Figure S3: Effect of OC-MQ-CM on CXCR1/2 expression in ovarian cancer cells; Table S1: List of oligonucleotide sequences; Table S2: Fold changes and *p*-values of MQ-CM-induced genes.
