# Supplementary material for: Enhanced Expression of TRIM46 in Ovarian Cancer Cells Induced by Tumor-Associated Macrophages Promotes Invasion via the Wnt/β-Catenin Pathway
Source: Cells. 2025 Feb 2;14(3):214. doi: 10.3390/cells14030214 (PMC11817100; doi:10.3390/cells14030214)
Supplement: Supplementary file 1 [file cells-14-00214-s001.zip › cells-3414525-supplementary.pdf]

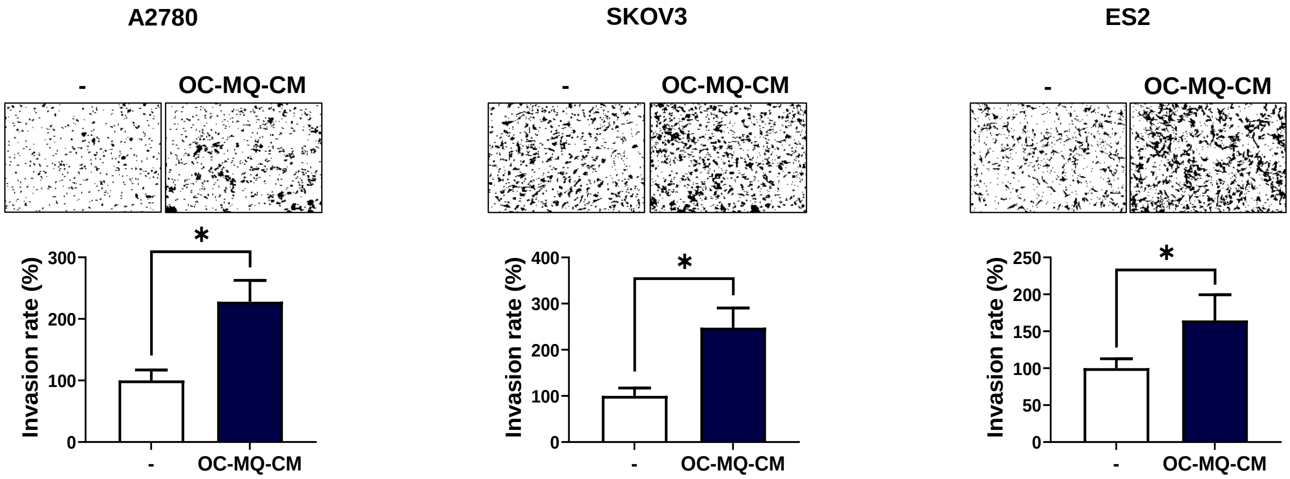

Supplementary Figure S1. Effect of OC-MQ-CM on ovarian cancer cell invasion

Ovarian cancer cells were resuspended in the CM of OC-MQ and seeded in Matrigel-coated upper chambers. The cells were allowed to invade for 24 hours. Results are representative of at least three independent experiments. *$p < 0.05$.

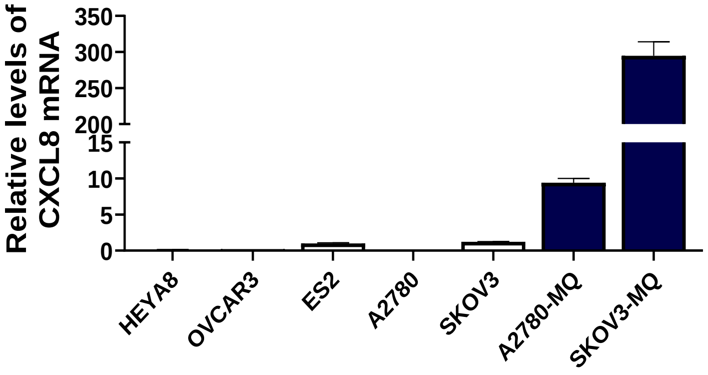

Supplementary Figure S2. Expression levels of CXCL8 in ovarian cancer cells and macrophages stimulated by ovarian cancer cells

Real-time RT-PCR was sued to measure the levels of CXCL8 in ovarian cancer cells (HEYA8, ES2, A2780, and SKOV3) and macrophages stimulated by A2780 or SKOV3 cells (A2780-MQ and SKOV3-MQ).

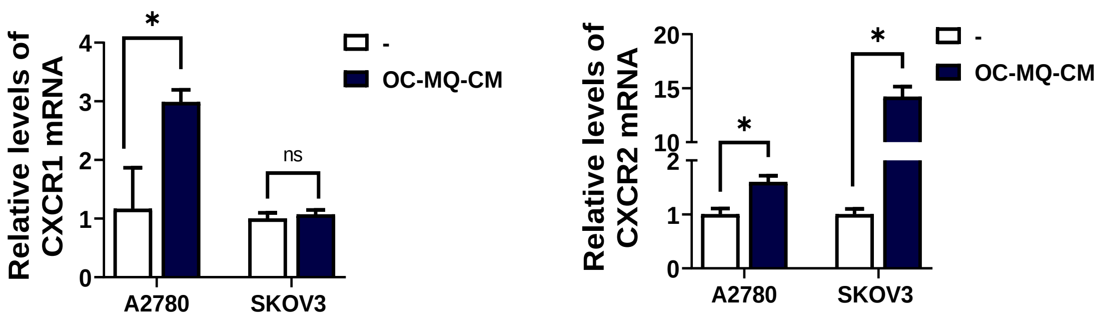

Supplementary Figure S3. Effect of OC-MQ-CM on CXCR1/2 expression in ovarian cancer cells

Ovarian cancer cells were treated with CM of OC-MQ for 24 hours. CXCR1/2 levels in A2780 and SKOV3 cells were examined using real-time RT-PCR $*p < 0.05$.
